# Diseases of the Lungs

**Published:** 1904-08-13

**Authors:** 


### DISEASES OF THE LUNGS.

Pneumonia.—As the result of an analysis of 70 cases of pneumonia treated in hospital, mainly among old people, Dawe and Austin[1] find a seasonal prevalence from March to June, a higher mortality among males than among females, and a localisation in right lower lobe 27 times, left lower lobe 11 times, right upper lobe six times, left upper lobe twice, whole right lung three times, whole left lung four times, and both lungs six times. Grey hepatisation was most frequent post-mortem in the cases of shortest duration, but more frequent than red hepatisation in those dying after the eighth day than before it. One death on the twentieth day showed red hepatisation. The condition had no relation to the age of the patient. The authors found the glands at the bifurcation of the trachea more frequently enlarged in pneumonia than in any disease except pulmonary tuberculosis. The heart was always dilated, chronic interstitial nephritis was the commonest co-existing lesion, and though delirium was common, no case of meningitis was seen. Phthisical patients did not seem predisposed to pneumonia. As to prognosis in old age (over 70) it is grave; in middle age it depends on the condition of the other organs, especially the kidneys; in youth it is good. A man of 72, however, recovered from a typical attack, followed by an empyema. They consider sweating at the height of the disease not uncommon, and a good sign; after the crisis, however, it denotes collapse, and is of bad import. They consider delirium increases the danger slightly in all cases, and considerably if mechanical restraint has to be used, or if it persists throughout the illness. Coma occurred in two cases as the initial symptom, and in two others terminally; all four died. Double pneumonia occurred in six cases with only one recovery—a teetotaller aged 44. Apical pneumonia did not appear especially dangerous or to be usually cerebral in type. A pulse-rate of above 120 was seldom seen in cases which recovered. Cases with a persistently low temperature sometimes ended fatally. Crisis occurred more than four times as often as lysis. The former usually took place on the seventh and ninth days, and the latter usually occupied three days. Atypical cases include those with coma, diagnosed as uræmia, those apparently mental (mania), and those presenting features of acute tuberculosis. In only one case was rusty sputum caused by another condition than pneumonia, viz., double hydrothorax with cirrhotic liver and kidneys. Reid[2] has recorded four cases with cerebral symptoms, one beginning with coma, one with transitory hemiplegia and hallucinations of vision, one with epileptic fits followed by two days' unconsciousness, and one in a known epileptic who died after two days of complete coma with tremors, frothing at the mouth, etc. All these cases occurred in adult women. Newton Pitt[3] has recorded the case of a man, aged 17, who was admitted into Guy's Hospital with intense dyspnœa, thoracic pain, noisy delirium, a temperature of 103·2, and a weak pulse of 120. No signs of consolidation of the lung were found, but signs of early peritonitis developed, and he died on the fourth day of his illness. Pleurisy was apparently the earliest lesion. Post-mortem there was lymph covering both lungs, commencing consolidation of the left apex, early pericarditis, endocarditis and peritonitis, blood-stained fluid in the ventricles, and enlarged mediastinal glands. The blood, pericardium, and peritoneum gave cultivations of the pneumococcus, and the case was a typical one of pneumococcal septicæmia, in which pneumonia had not had time to develop. With regard to the treatment of pneumonia, Thompson[4] considers that no specific treatment exists, that creosote is not of value, and that alcohol should only be given in moderate doses when the pulse is feeble or the patient very weak, aged, or alcoholic. Expectorants he considers contra-indicated. The condition of the stomach and bowels he thinks very important, as abdominal distension embarrasses the chest, and auto-intoxication results from indigestion. Calomel, followed by non-effervescent salines, should be used. Hiccough can be relieved by anodynes, but is a dangerous condition. Morphia is dangerous, but with hyoscine hydrobromide and paraldehyde may be tried in severe alcoholic delirium. Oxygen may be useful early in the case, and fresh air and hypodermic injections of saline solutions are veiy valuable. Heat locally is generally more soothing than cold, but neither has more than a subjective effect on the patient. Milk should be given freely, the mouth kept sweet and clean, and over-stimulation and expectorants avoided. Good intervals of rest should always be allowed, as sleep is most important. Peabody[5] agrees that oxygen must be given early, that the disease cannot be aborted, and that hypnotics are inadvisable; nevertheless, that morphine in small doses subcutaneously, or paraldehyde or amylene hydrate (per rectum or in peptonised milk) often do good. Though antiseptics are useless, he believed salicylates or aspirin are valuable, producing in many cases a rapid termination by lysis. Conner[6] deprecates the use of large amounts of alcohol, and emphasises the importance of watching the abdomen; tympanites he thinks best controlled by turpentine stupes and irrigation of the colon alternately with hot and cold water. Pulmonary œdema yields to frequent dry cupping. Quimby[7] believes that topical applications do good, and that the diminution of chlorides in the urine is an indication for sodium chloride from the beginning. Menges[8] also considers the abdominal condition of great importance; he considers morphine and cathartics both dangerous, but favours dry cupping and venesection. He thinks guiacol especially useful in influenzal cases, and the salicylates in children. Lambert[9] has found ergot useful both for the tympanites and for œdema of the lungs in conjunction with dry cupping. Compton[10] divides remedial agents into fundamental (influencing the toxæmia)

and accessory (temporarily stimulating the patient). The most important of the former class is hypodermoclysis, which he first introduced to dilute toxins and preserve the alkalinity of the blood. Eight ounces to one pint should be injected daily as occasion demands. Of less value are quinine hypodermically and the carbonates of guiacol and creosote. The accessory agents are alcohol, morphia, digitalis, strychnine, etc. Altshul[11] claims a specific action for large doses of iodide of potassium—500 to 3,000 grains per diem. It is rapidly absorbed and diffused, and may be given by the mouth, rectum, subcutaneous tissue, veins, skin, or muscles. It acts as an antiseptic by means of the iodine liberated in the blood; it increases leucocytosis, which is admittedly a favourable reaction in pneumonia; it increases the alkalinity and lowers the specific gravity of the blood, both of which effects have been shown to be of value in the pneumococcic septicæmias of animals; it is a vaso-dilator, and thus, like the nitrites, obviates asphyxia, and directly stimulates the heart by increasing its blood supply; it is a diuretic, diaphoretic, antipyretic, and respiratory sedative. In practice he has frequently observed lysis termination under the drug, lowering of the temperature, absence of complications and cerebral symptoms, and has found so marked a toleration of large doses that he concludes that a specific action must be present. It may usually be given in 50 per cent. solution in milk. Treatment should be guided by the condition of the heart, and especially the distinctness of the pulmonary sounds. Renzi's *Siero anti-pneumonico* has been used with good effect by Rentoul[12] in a case when ordinary treatment had failed, and the patient on the fourth day appeared moribund. The pulse and respiration rate fell within four hours of the injection, and the sputum became less blood-stained. A second injection next day was followed by a marked fall of temperature, and convalescence was rapid.

Spasmodic Asthma.—Two main theories have been propounded to account for the paroxysms of this disease. Both admit that it is an exaggeration of a normal reflex, hereditary and peculiar in certain individuals. Wilkinson[13] regards the reflex arc as beginning in some part of the skin or mucous membrane, travelling up to centres in the medulla, and then down branches of the vagus, producing constriction of the bronchioles—or in the catarrhal form passing down to the mucous surfaces of the nose and pharynx. He cites experimental evidence to prove that constriction of the bronchioles can occur, and considers the results of medication support his view. Hare,[14] on the other hand, attributes the bronchial symptoms to vaso-dilatation of the pulmonary mucous membrane, accompanied either causally or secondarily by general vaso-constriction. He quotes in support the analogy of migraine and urticaria, and the results of one post-mortem examination made by Fränkel. The effects of drugs he also considers support this theory rather than the other. As to treatment, Wilkinson points out that a constitutional tendency or habit being present remedial measures must be long continued. He divides exciting causes into aerial and particulate. The former include the emanations from certain animals of which he quotes examples. These give rise to bronchial asthma, and must be first ascertained and removed. The latter are more apt to cause symptoms of coryza. Among drugs he strongly advocates arsenic and potassium iodide, the latter, however, is transitory in effect and the symptoms recur when it is discontinued. Both these drugs, he thinks, dilate the bronchioles. Belladonna he thinks also useful, but dangerous in old people with secondary bronchitis as preventing the expectoration of the secretions. Nitrites he considers disappointing on the whole. Naso-pharyngeal obstruction and digestive disturbances must be remedied, acting as they do on the sensory end of the reflex arc. He considers the treatment of the paroxysm by fumigations justifiable and less likely to damage the heart than long and frequent attacks. He cautions practitioners against hypodermic injections of morphia, chloroform inhalations, and the use of alcohol and cocaine as likely to produce a most disastrous drug habit. Hare considers that potassium iodide acts partly by promoting bronchial secretion, and recommends breathing cold air, inhalation of nitre and other fumes, and painting the inferior turbinates with chromic acid in order to produce local vaso-constriction, or conversely amyl nitrite, nitroglycerine, stramonium, belladonna, alcohol, and opium to produce general vaso-dilatation in other areas. Hot applications to the chest and dry cupping, are also useful; ipecac, lobelia, tartar emetic, and tobacco may be employed to lessen cardiac action, and venesection to produce a sudden fall of blood pressure. Dresler[15] recommends Beyer's aristochin in doses of six grains thrice daily, which he has used successfully after other remedies had failed. The only ill effects were slight pruritus and tinnitus sometimes observed. Campbell[16] for nine years has employed intratracheal injections of menthol, glycerine and gelatin. Thirty grains of menthol to two ounces of glycerine may be used. The menthol, he considers, relieves the bronchial spasm, the glycerine stimulates secretion, and the gelatin softens it, the two theories as to the causation of asthma being thus equally acted upon.

[1] Lancet, Feb. 20, 1904, p. 496. [2] Brit. Med. Jour, April 30, 1904, p. 1014. [3] Ibid, March 19, 1904, p. 665. [4] Med. Rec., March 12, 1904, p. 434. [5] Ibid., p. 435. [6] Ibid. [7] Ibid. [8] Ibid., p. 436. [9] Ibid. [10] Med. Chron., Feb. 1904, p. 301. [11] Med. Rec., March 26, 1904, p. 481. [12] Brit. Med. Jour., March 19, 1904, p. 663. [13] Med. Chron., Jan. 1904, p. 219. [14] Med. Rec., Feb. 27, 1904, p. 341. [15] Therap. der Gegenwart, Dec. 1903. [16] Liverpool Med. Chir. Journ., Jan., 1904.

